# Influence of Infiltration Pressure on the Microstructure and Properties of 2D-CFRP Prepared by the Vacuum Infiltration Hot Pressing Molding Process

**DOI:** 10.3390/polym11122014

**Published:** 2019-12-05

**Authors:** Yuqin Ma, Yatao Zhao, Yun Zhang, Jie Wang, Yi Chen, Kaifu Li, Luyan Ju, Ying Yu

**Affiliations:** 1 School of Mechano-Electronic Engineering, Xidian University, Xi′an 710071, China; xidianyatao@163.com (Y.Z.); yunzhang@xidian.edu.cn (Y.Z.); imustwj@163.com (J.W.); xdchenyi@163.com (Y.C.); Leekaifu521@163.com (K.L.); yuying_1114@163.com (Y.Y.); 2 Mechanical engineering college, Xi′an Shiyou University, Xi′an 710065, China

**Keywords:** infiltration pressure, vacuum infiltration hot pressing molding process, 2D-CFRP, microstructure and properties, influence

## Abstract

The critical infiltration pressures of the matrix in a two-dimensional (2D) carbon fiber preform were calculated theoretically, and the calculated values of the static and dynamic models were 0.115 and 0.478 MPa, respectively. Compared with the dynamic model, there is no viscous resistance or infiltration front gas pressure in the static model, so the static value is obviously lower than the dynamic value. To verify the rationality of theoretical calculation, 2D carbon fiber reinforced plastics (2D-CFRP) with infiltration pressures of 0.5, 0.6, 0.7, 0.8, and 0.9 MPa were prepared by the vacuum infiltration hot pressing molding process. The microstructure of the composite was observed and the bending strength was tested by three-point bending test. The results show that the infiltration pressure has an important influence on the infiltration effect and the bending fracture morphology. When the infiltration pressure is 0.7 MPa, the composite has an excellent infiltration effect. The fibers distribute reasonable in the fracture. Stress can be effectively transferred when the composite material is loaded. And the bending strength of the composite material reaches 627 MPa at this time.

## 1. Introduction

Carbon fiber reinforced resin matrix composite has a series of outstanding properties, such as low density, high specific strength, high specific modulus, chemical corrosion resistance, high thermal conductivity, and low thermal expansion coefficient [1,2]. It is widely used in aerospace, military, and automobile industries, electronics, and civil engineering [3,4]. The infiltration process is one of the most important steps in preparing2D-CFRP by the vacuum infiltration hot pressing molding process. To prepare 2D-CFRP with excellent properties, the matrix needs be sufficiently and uniformly infiltrated in the 2D carbon fiber preform. Otherwise, there will be pores or local non-infiltration among the carbon fiber bundles, and carbon fibers cannot play the role of reinforcement effectively, which will greatly affect the microstructure and properties of 2D-CFRP [5]. The experimental results show that the infiltration effect of matrix in a 2D carbon fiber preform is partly determined by the infiltration pressure. If the infiltration pressure is too low, the matrix in the 2D carbon fiber preform is not sufficient and uniform. If the infiltration pressure is too high, the composite will produce cracks easily. Therefore, it is significant to study the influence of infiltration pressure on the microstructure and properties of 2D-CFRPprepared by the vacuum infiltration hot pressing molding process.

At present, some experts and scholars at home and abroad have carried out relevant studies on the infiltration pressure laws of composite materials and achieved certain results. Venugopalanet al. [6] used the liquid phenolic resin matrix to infiltrate 2D preform. Their study showed that with the increase of infiltration pressure, the porosity of the composite decreased, and the combination of liquid phenolic resin matrix and preform improved to some extent under high infiltration pressure, while the infiltration time had little influence on the porosity. Nasur et al. [7,8], after analyzing the infiltration behavior of different metal materials, obtained the critical infiltration pressure laws of liquid metal infiltrate fiber preform from the fluid theory level. Guan Juntao et al. [9] designed a dynamic measuring device to analyze the process of liquid metal infiltrate fiber preform. In the experiment of magnesium alloy infiltrate fiber preform, the critical infiltration pressure of liquid metal infiltrate fiber preform was calculated and measured. Wannasinet al. [10] used the high-pressure centrifugal infiltration method to measure the critical infiltration pressure of compacted ceramic particles infiltrate tin lead alloy with different volume fractions, and established a model to predict the critical infiltration pressure. Liu HN et al. [11] studied the axial infiltration process of Al_2_O_3_ continuous fiber reinforced Al-Cu alloy in depth. By studying the infiltration process, the equations for calculating the critical infiltration pressure and defects size of composite materials were derived. However, the study on the critical infiltration pressure of the matrix in a 2D carbon fiber preform is not perfect at present.

This paper adopts the method of combining theories and experiments, and analyzed the infiltration process from statics and dynamics by establishing the model of infiltration mechanics. By calculating the static and dynamic model values of the matrix infiltrating the 2D carbon fiber preform were 0.115 and 0.478 MPa, respectively. On this basis, the influence of the infiltration pressures within a range of 0.5–0.9 MPa on the infiltration effect and properties were studied experimentally. Finally, the theoretical and experimental critical infiltration pressures of matrix in the 2D carbon fiber preform were obtained, which laid a foundation for preparing2D-CFRP with an ideal microstructure and properties.

## 2. Experimental Materials and Methods

### 2.1. Experimental Materials

The matrix used in the experiment was E-44 epoxy resin produced by the Xi′an Resin Factory in Xi′an, China, and the curing agent was 593 curing agent produced by Sanmu Group Co. LTD in Yixing, China. The reinforcement was 2D-T300 carbon fiber produced by Toray Company in Tokyo, Japan. The instruments and equipment used include a JEOL JSM-6390A scanning electron microscope (SEM), vacuum oven, thermo press machine, bending performance measurement equipment, electronic balance, etc. The SEM is produced by Japan Electronics Co., Ltd in Tokyo, Japan. The vacuum oven is produced by Beijing Kewei Yongxing Instrument Co., Ltd in Beijing, China. The thermo press machine is made by Hebi Xinge Instrument Sales Co., Ltd in Hebi, China. The bending performance measurement equipment is made by Changchun Institute of Mechanical Science in Changchun, China. The electronic balance is produced by Changshu Shuang jie test instrument factory in Changshu, China.

### 2.2. Experimental Methods

**Figure 1:**
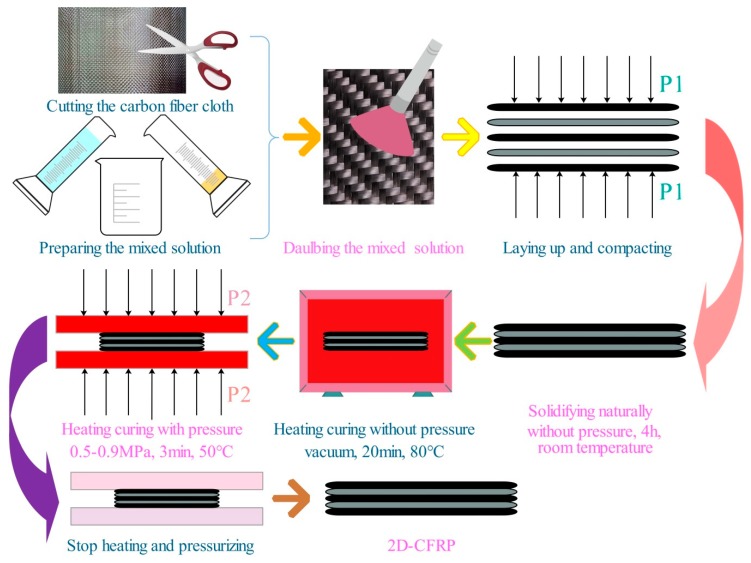
Process of preparing a 2D-CFRP composite.

In the experiment, 2D-T300/E44 composite material was prepared by the vacuum infiltration hot pressing molding process. The detailed steps to prepare 2D-CFRP as follows: (1) 2D-T300 carbon fiber cloths are cut and the size is 60 mm × 60 mm. The curing mixed solution of epoxy resin is prepared and the mass ratio is 5:1. (2) The prepared mixed solution is applied uniformly on the two sides of the cut 2D-T300 carbon fiber cloths, and the carbon fiber cloths are laid down and stacked in order with a thickness of 2 mm. Then a pressure is applied to the carbon fiber laminate for pre-compaction. (3) The prepared carbon fiber laminate is placed in room temperature for naturally curing for four hours. Then it is transferred to a vacuum oven (−0.09 MPa) of constant temperature drying, the curing time controlled for 20 min, and the temperature controlled at 80 °C. (4) The carbon fiber laminate is taken out from the vacuum oven of constant temperature drying and is put into the preheated hot-pressing mold. At 50 °C, the infiltration pressures in each experiment are 0.5, 0.6, 0.7, 0.8, and 0.9 MPa, respectively, and the pressure is maintained for 3 min. (5) After the heating and pressure stop, the prepared 2D-T300/E44 composite is taken out after the mold is restored to room temperature. The process of preparing 2D-T300/E44 composite by the vacuum infiltration hot pressing molding process is shown in Figure 1.

#### 2.2.1. Vacuum Infiltration Hot Pressing Molding Process

The vacuum infiltration hot pressing molding process contains two processes, namely vacuum infiltration and hot pressing molding, whose essence is to assist the matrix infiltrate the preform and complete the curing process with the help of vacuum, high temperature and pressure. In the process, vacuum infiltration refers to placing the carbon fiber laminate coated matrix in a constant temperature (80–120 °C) vacuum oven. With the aid of vacuum environment and high temperature, it is helpful for accelerating the preform internal gas discharge. This is more conducive for the matrix infiltrates the fiber preform and avoids defects. Hot pressing molding shows that the vacuum-infiltrated carbon fiber laminate is moved into the preheated mold, which is heated to 100–130 °C and then cooled to 50 °C to conduct the hot pressing molding curing process. Mechanical pressure of 0.5–10 MPa is applied to accelerate the flow of the matrix in the preform so that the matrix can infiltrate into the gap that failed to complete the infiltration before and, in a timely manner, make up for shrinkage under high pressure to reduce the shrinkage cavity and porosity and other defects [12]. Compared with the traditional infiltration process, the vacuum infiltration hot pressing molding process can greatly improve the infiltration effect of matrix in preform and reduce the usual defects in preparing composites, which is of great significance for improving the properties of 2D-CFRP products.

#### 2.2.2. Process Parameters

Adopting the above process to prepare 2D-CFRP involves the curing mixture ratio, pressure, temperature, and time. When the process parameters selected are not proper, the prepared 2D-CFRP will easily suffer defects, which will have an undesirable impact on the microstructure and properties of 2D-CFRP. According to the properties of matrix and previous experimental experience, the detailed process parameters are shown in Table 1.

(1) Static Analysis and Calculation of Infiltration Pressure

**Figure 2:**
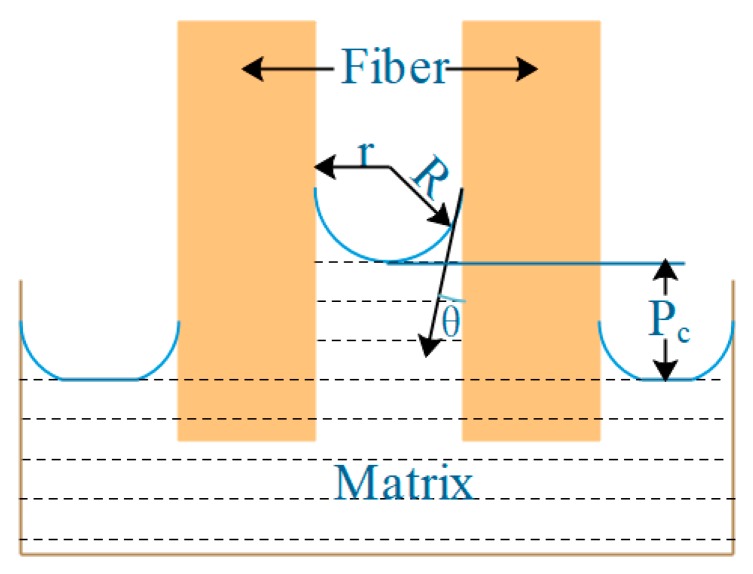
Schematic diagram of capillary pressure difference.

2D carbon fiber preform can be regarded as a porous medium when infiltrated by matrix. The pressure difference generated by capillary action in 2D carbon fiber preform determines whether the infiltration can occur spontaneously. The pressure difference generated by capillary action is shown in Figure 2. The value can be calculated by the Yong–Kelvin Equation [13]:

(1) $$P_{c}=\frac{2\gamma_{1v}cos\theta }{r}$$

where *P*_c_ (MPa) is capillary pressure difference; γ*_lv_* (N/m) is the surface tension of the liquid; θ (°) is the wetting angle between liquid and solid; and *r* (um) is the capillary radius.

**Figure 3:**
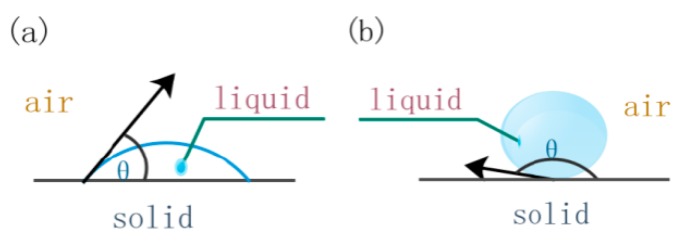
Two conditions of liquid on solid surface: (**a**) Wetting condition of liquid on solid surface; (**b**) not wetting condition of liquid on solid surface.

The Yong–Kelvin Equation shows that, when θ < 90°, *P*_c_ > 0, the liquid can wetting the solid, as shown in Figure 3a, and the infiltration can occur spontaneously. When θ > 90°, *P*_c_ < 0, the liquid cannot wetting the solid and the infiltration cannot occur spontaneously [14], as shown in Figure 3b. In this case, external pressure is required to overcome the capillary pressure infiltration.

**Figure 4:**
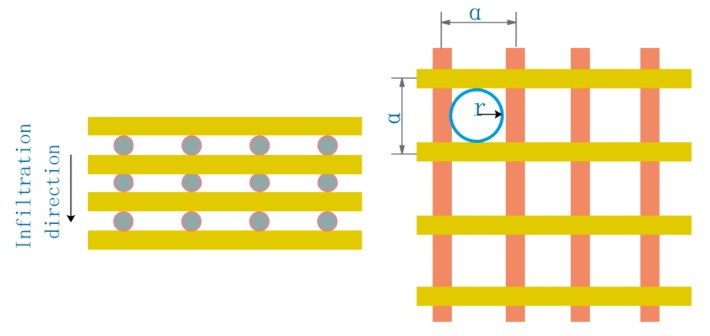
Distribution model.

For systems that need to apply external pressure, the minimum external pressure required to start infiltration is called the critical infiltration pressure *P*_th_, and its main form is capillary pressure *P*_c_. Since the wetting angle between the matrix and the 2D carbon fiber preform is less than 90°, the infiltration can occur spontaneously in theory. Due to the slow reaction rate, the infiltration effect easily appears insufficient and non-uniform and external pressure is still needed to assist the infiltration. At this time, the calculation equation of the critical infiltration pressure *P*_th_ can be calculated by Equation (2):

(2) $$P_{th}=P_{v}-\frac{2\gamma_{lv}cos\theta }{r}$$

where *P*_th_ (MPa)is the critical infiltration pressure, *P*_v_ (MPa) is the gas pressure in the preform of the infiltration front fiber. In the experiment, the infiltration was carried out under a vacuum condition, so *P*_v_ is 0. *ρ* is the density of the liquid, *h*_0_ is the height of the liquid, and $\rho gh_{0}$ is very small, which can be ignored.2D-T300 carbon fiber sheets are laid out in a plane layer, which accords with the approximate calculation model of the plane distribution of fibers. As shown in Figure 4, the equivalent radius used in calculating capillary pressure should be calculated according to the following Equation [15].

(3) $$\{\begin{array}{c} \;a=\frac{\pi d_{f}}{4V_{f}}\\ r\approx \frac{1}{2}(a-d_{f})\\ =\frac{1}{8V_{f}}\left(\pi -4V_{f}\right)d_{f} \end{array}$$

During the experiment, the fiber volume fraction *V*_f_ is 65%, and the wetting angle between matrix and carbon fiber θ is 12°.The fiber capillary radius is calculated from Equation (3) *r* = 0.73 μm. Surface tension γ*_lv_* is 0.043 N/m and the average diameter of fiber *d*_f_ is 7μm. By substituting the above parameters into Equation (2), the critical infiltration pressure calculated by static model is about 0.115 MPa.

(2) Dynamic Analysis and Calculation of Infiltration Pressure

**Figure 5:**
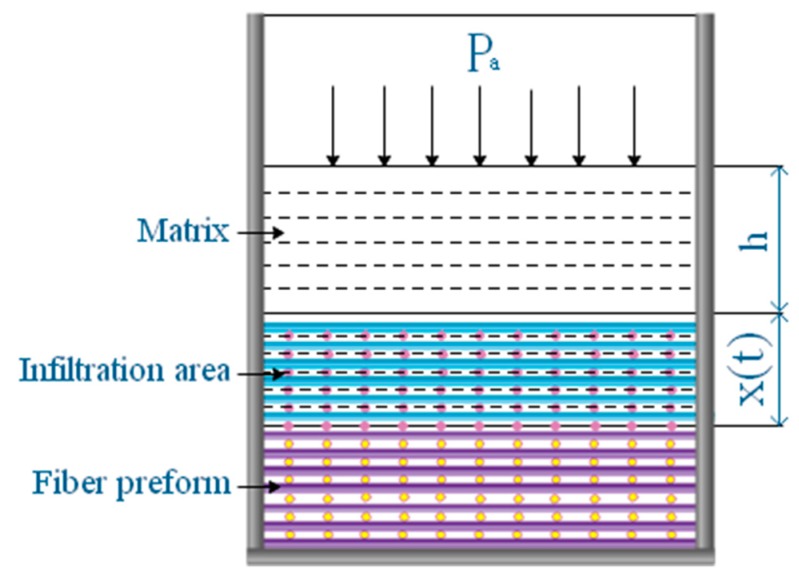
Schematic diagram of the infiltration process of matrix into a 2D carbon fiber preform.

The infiltration process of matrix in 2D carbon fiber preform is shown in Figure 5. In the process of infiltration, the resistance of matrix increases with the deepening of infiltration. For more aptly describe the actual situation of matrix infiltrates 2D carbon fiber preform, from the angle of dynamic analysis matrix and infiltration of the dynamic flow of resistance, and at the same time to reduce the influence factors to ensure the experimental rigor, for the following assumptions. (1) The 2D carbon fiber preform and the hot pressing mold have been preheated before the infiltration. The effect of heat conduction during the infiltration is ignored. It is also assumed that the matrix is at a constant temperature during the infiltration process. (2) Matrix flows steadily in the capillary, and the influence of the resistance at the front end is ignored. (3) The 2D carbon fiber preform has an internal vacuum and the opposite pressure of gas on the matrix is ignored [9].

The matrix is mainly affected by the capillary pressure, viscous resistance and air resistance during the infiltration process. In the experiment, the infiltration process is carried out under a vacuum environment, so there is no need to consider the influence of air resistance. For the preform with uniform fiber distribution, the capillary pressure *P*_th_ can be calculated by Equation (4) [13]:

(4) $$P_{c}=\frac{4cos\theta \sigma_{1g}V_{f}}{d_{f}\left(1-V_{f}\right)}$$

where *V*_f_ (%) is the fiber volume fraction; $\sigma_{1g\;\;}$ (N/m) is the liquid surface tension; θ (°) is the wetting angle; and *d*_f_ (μm)is the fiber diameter. By substituting the relevant parameters into Equation (4), the capillary pressure generated by matrix in the infiltration process is calculated to be about 0.04 MPa.

When the liquid matrix flows in the form of laminar flow in the 2D carbon fiber preform, it is affected by viscous resistance. The pressure drop generated by the viscous resistance can be calculated by Equation (5) [16]:

(5) $$△p=\mu (1-V_{f})h^{2}/2Kt$$

where *μ* is the dynamic viscosity, h is the infiltration height, *K* is the permeability coefficient, t is the infiltration time. The dynamic viscosity of the matrix is 0.562 Pa/s^−1^, and the infiltration height *h* is set at 2 mm. It has been assumed that the infiltration time is short at 40 s. The permeability coefficient can be calculated by Blake–Kozeny Equation (Equation (6)):

(6) $$k_{bk}=\frac{d_{f}^{2}(1-V_{f}{)}^{3}}{150V_{f}}$$

By Substituting *V*_f_ and *d*_f_ into Equation (6), the permeability coefficient *K* can be calculated is $1.9\times 10^{-14}{\;m}^{2}$. By substituting the permeability coefficient into Equation (5) the viscous resistance can be calculated is 0.5176 MPa. The external pressure required for infiltration Equation (7) can be obtained from Equations (4) and (5):

(7) $$P=-\frac{4cos\theta \sigma_{1g}V_{f}}{d_{f}\left(1-V_{f}\right)}+\frac{\mu (1-V_{f})h^{2}}{2Kt}$$

By substituting the previous calculation results into the equation, it is obtained that the applied critical infiltration pressure required for the preparation of 2D-CFRP by dynamic analysis is 0.478 MPa.

Although the capillary pressure and viscous resistance are taken into consideration in the dynamic model, it is still based on an idealized infiltration model. The influence of air resistance and front end resistance are ignored. Therefore, the above calculation and analysis are only regarded as a theoretical guidance. In the actual process of infiltration, only when the infiltration pressure is greater than 0.478 MPa, the matrix can be infiltrated in a 2D carbon fiber preform spontaneously.

#### 2.2.3. Testing and Characterization Methods

**Figure 6:**
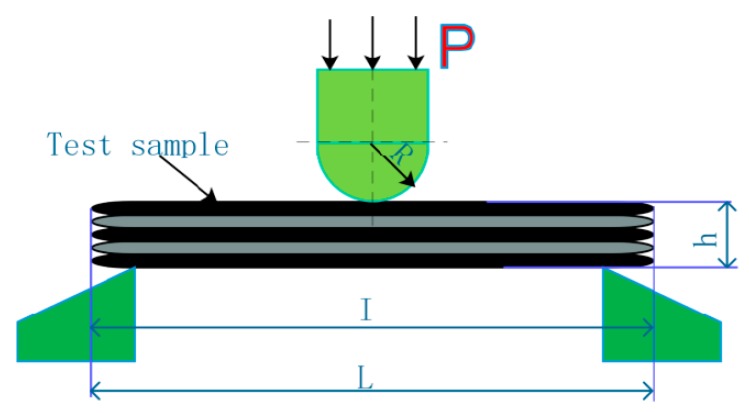
Three-point bending test method schematic diagram. *P*: Applied load. R: Radius of the upper pressure head under loading. *H*: Specimen thickness. *I*: Span of sample. L: Sample length.

The infiltration microstructure and bending fracture morphologies of 2D-T300/E44 composite were observed by a JEOLJSM-6390A SEM. The three-point bending strengths of 2D-T300/E44 composite were tested by a DNS100 electronic universal testing machine at the Changchun Institute of Mechanical Science in Changchun of China and the specific test methods are in accordance with the Test Method for Flexural Properties of Fiber-Reinforced Plastics (GB/T1449-2005). The size of the bending samples is 50 mm × 15 mm × 2 mm with the span of 40 mm. The load on the pressure head radius *R* is 5 mm and the test loading speed is 10 mm/min. The test schematic is shown in Figure 6.

## 3. Experimental Results and Discussion

The infiltration pressure applied during the infiltration process was changed successively on the basis of the theoretical derivation and calculation. The infiltration effect of matrix in the 2D carbon fiber preform under different infiltration pressures was observed. The bending strengths of 2D-CFRP prepared under different infiltration pressures were measured. It is concluded that the infiltration pressure has a vital influence on the infiltration effect. Low infiltration pressure will lead to insufficient and non-uniform infiltration, while high infiltration pressure will lead to cracks in the microstructure. Therefore, in order to prepare 2D-CFRP with ideal microstructure and excellent properties, it is necessary to study the influence of different infiltration pressures on the microstructure and properties of 2D-CFRP.

### 3.1. Influence of Infiltration Pressure on the Preparation of 2D-CFRP

**Figure 7:**
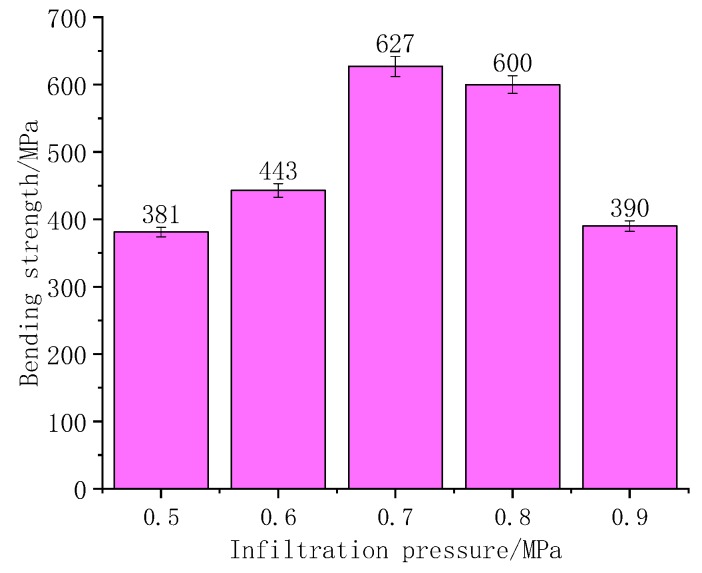
Comparison of bending strengths of CFRP composite under different infiltration pressures.

The critical infiltration pressure calculated by the dynamic is 0.478 MPa. Considering that the influence of the air resistance and front end resistance was ignored in the calculation, thus, the initial infiltration pressure in the experiment was set at 0.5 MPa, and increases the infiltration pressure with the results of the experiment in turn. The different infiltration pressures of 2D-CFRP, respectively, are observed by the three-point bending experiment method in measuring the bending strengths, with specific results as shown in Figure 7.

(1) The Infiltration Pressure of 0.5 MPa

**Figure 8:**
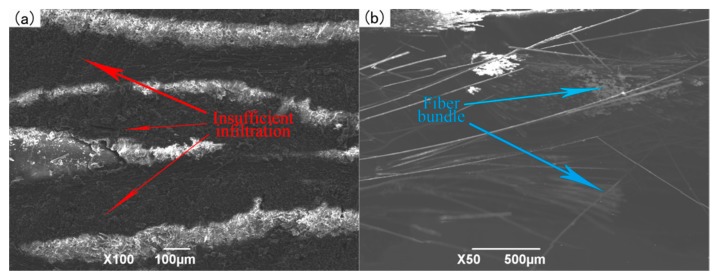
Micrographs of 2D-CFRP (0.6 MPa): (**a**) Infiltration microstructure (100×); (**b**) shape diagram of bending fracture (50×).

**Figure 9:**
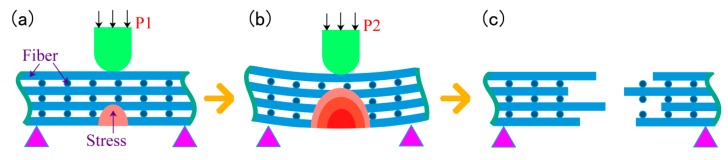
Schematic diagram of fracture process and stress distribution (0.5 MPa).

When the infiltration pressure is 0.5 MPa, the microstructure of the 2D-CFRP scanned by SEM is shown in Figure 8a and the bending fracture morphology is shown in Figure 8b. It can be seen from Figure 8a that the fiber bundle is dark and the matrix is grey. At this time, there is small amount of matrix distributed inside the 2D carbon fiber preform, but the infiltrated areas are still slightly dark, which shows that the content of matrix is low. Therefore, a small amount of matrix cannot bind all fiber bundles together. The infiltration effect is insufficient and non-uniform at this time. In the experiment, the main reason is that the infiltration pressure is low [17]. It can be seen from Figure 8b that the fracture is dark in color, with only a small amount of matrix scattered among the fiber bundles and a large number of fibers scattered among the uniform fracture. The fracture morphology is shown in Figure 9c, and there is no matrix on the broken fibers. When the composite material is loaded, a small amount of matrix cannot play the role of load transfer, and the stress is mainly borne by few fibers. The stress distribution is shown in Figure 9a,b. The bending strength is relatively low in theory, and the fracture process and stress distribution are shown in Figure 9.

When the infiltration pressure is 0.5 MPa, the content of matrix inside the microstructure is less, and carbon fiber cannot strengthen the properties as well as it could, which leads to the poor bending performance of the prepared 2D-CFRP. Through the three-point bending test, the bending strength is only 381 MPa. At this time, the infiltration pressure should be appropriately increased to further observe the infiltration effect of matrix in 2D carbon fiber preform and the change of the bending property of the composite.

(2) The Infiltration Pressure of 0.6 MPa

**Figure 10:**
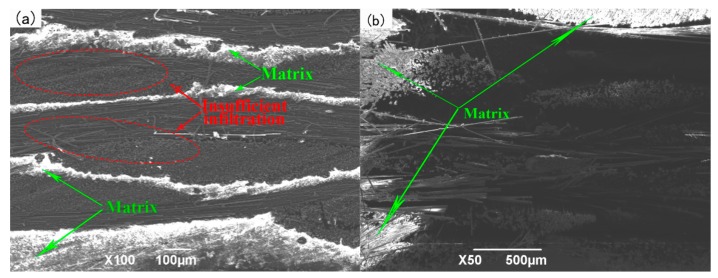
Micrographs of 2D-CFRP (0.6 MPa): (**a**) Infiltration microstructure (100×); (**b**) shape diagram of bending fracture (50×).

When the infiltration pressure is increased to 0.6 MPa, the microstructure of 2D-CFRP scanned by the SEM is shown in Figure 10a and the bending fracture morphology is shown in Figure 10b. It can be found from Figure 10a that a large amount of matrix distributes among fiber bundles, and the infiltrated areas are whiter and brighter compared with the infiltration pressure of 0.5 MPa, but the matrix still has not completely infiltrated in the 2D carbon fiber preform. The middle part of the matrix is long and thin strip, and the content of matrix is not very full, which shows that the infiltration pressure is still low. It can be seen from Figure 10b that matrix is not evenly distributed at the fracture of the prepared 2D-CFRP. The distribution of fibers is more reasonable and the fracture is relatively orderly compared with the infiltration pressure of 0.5 MPa. However, there are still some areas with low matrix content at the fracture, and there is only a small distribution of matrix on the broken fiber. Though the overall matrix content increases, the matrix content is still not sufficient. This leads to the failure of effective stress transfer in 2D-CFRP.At this time, the overall infiltration effect and mechanical properties of the composite material has been improved, but it is still not ideal.

When the infiltration pressure is 0.6 MPa, the content of matrix improves significantly. However, the matrix still failed to bind the carbon fiber together, only part of the carbon fiber can play the role of reinforcement. The bending strength of the composite material reaches 443 MPa, which has significant improvement, and there is still possibility to improve the infiltration effect and composite properties. At this time, the infiltration pressure should be increased appropriately to observe the infiltration effect of matrix in the 2D carbon fiber preform and the change of the bending property of the composite material.

(3) The Infiltration Pressure of 0.7 MPa

**Figure 11:**
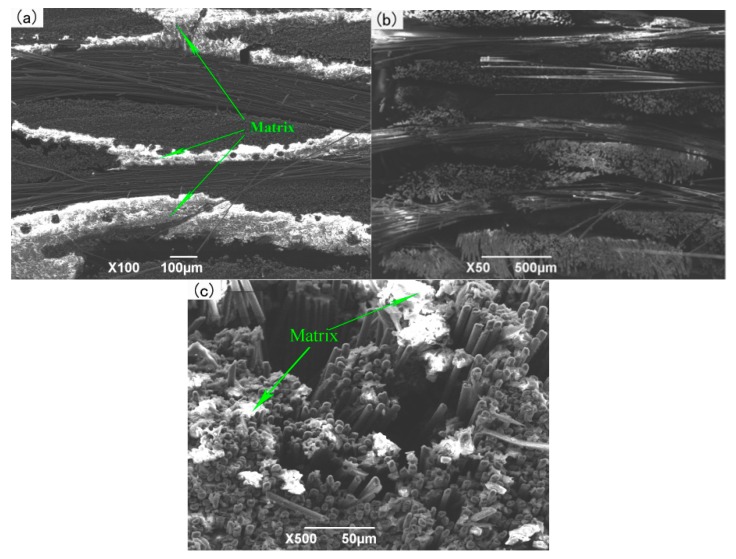
Micrographs of 2D-CFRP (0.7 MPa): (**a**) Infiltration microstructure (100×); (**b**) shape diagram of bending fracture (50×); (**c**) shape diagram of bending fracture (500×).

**Figure 12:**
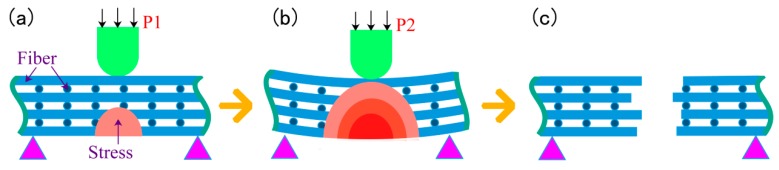
Schematic diagram of fracture process and stress distribution (0.7 MPa).

When the infiltration pressure is further increased to 0.7 MPa, the microstructure of the 2D-CFRP scanned by SEM is shown in Figure 11a and the morphologies of bending fracture is shown in Figure 11b,c. It can be seen from Figure 11a that there is a large amount of matrix distributes among fiber bundles. The color of the infiltrated areas is whiter and brighter than before, and the infiltration effect is fine at this time. It can be seen from Figure 11b that there is a large amount of matrix inside the fiber bundles at the fracture. The distribution of carbon fiber and matrix is more reasonable. Further magnifying the fracture microstructure, it can be seen from Figure 11c that the matrix inside the microstructure is significantly increased and more evenly distributed, and the fibers are closely wrapped by matrix. The composite material fracture is neat, and part of the fiber is pulled off and part of the fiber is pulled out. The number and length of the pulled out fibers are considerable. The results show that the cracks are effectively bifurcated and deflected in the interfacial phase, which makes the cracks front stress effectively dispersed. The schematic diagram of stress distribution is shown in Figure 12a,b. Therefore, the fracture mode of matrix can be determined as ductile fracture [18]. At this time, carbon fiber can effectively play the strengthening role, and the matrix can also play an effective role in transferring load. The fracture process diagram and stress distribution are shown in Figure 12.

When the infiltration pressure is 0.7 MPa, the bending property of the 2D-CFRP is further improved, and the bending strength reaches 627 MPa, which indicates that the property of the composite material is relatively ideal. In order to determine the optimal infiltration pressure, the infiltration pressure should be appropriately increased to further observe the infiltration effect of matrix in the 2D carbon fiber preform and the change of bending properties of the composite material.

(4) The Infiltration Pressure of 0.8 MPa

**Figure 13:**
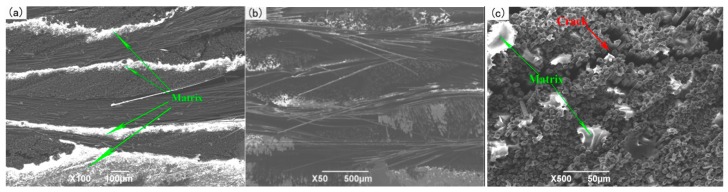
Micrographs of 2D-CFRP (0.8 MPa): (a) Infiltration microstructure (100×); (b) shape diagram of bending fracture (50×); (c) shape diagram of bending fracture (500×).

When the infiltration pressure is 0.8 MPa, the microstructure of the 2D-CFRP scanned by SEM is shown in Figure 13a and the bending fracture morphologies are shown in Figure 13b,c. It can be seen from Figure 13a that there is amount of matrix distributed among the fiber bundles. The matrix is also evenly distributed. It can be seen from Figure 13b that there is a large amount of matrix adhering to the fibers at the fracture. The fiber distributes relatively neat and reasonable in the fracture, and the infiltration effect is good. Further magnifying the microstructure at the fracture, it can be seen from Figure 13c that the matrix is sufficiently and uniformly distributed in the fibers, and the fracture morphology is relatively neat. Most of the fibers are pulled off and a few fibers are pulled out, but there is a significant crack in the fracture. Considering that the composite material has a good infiltration effect, during the bending test the load on the crack will be transferred to the adjacent area through the matrix so as to prevent the occurrence of interlocking fracture [19]. Therefore, the bending strength of the 2D-CFRP prepared will be slightly lower than 627 MPa theoretically.

When the infiltration pressure is adjusted to 0.8 MPa, the bending strength of the prepared 2D-CFRP reaches 600 MPa, which is slightly lower than the 2D-CFRP prepared under the infiltration pressure of 0.7 MPa. The above theoretical analysis is also verified by the fact that the slightly higher infiltration pressure not only affects the infiltration effect, but also brings some defects to the 2D-CFRP.Sincethe infiltration effect and bending strength of composite materials do not change a great deal, it is necessary to further improve the infiltration pressure and observe the infiltration effect and the change of the bending property of the composite material.

(5) The Infiltration Pressure of 0.9 MPa

**Figure 14:**
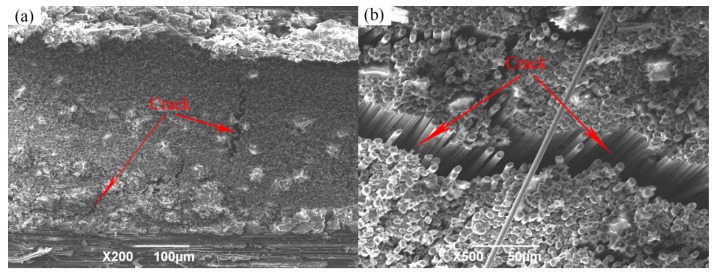
Micrographs of 2D-CFRP (0.9 MPa): (**a**) Infiltration microstructure (200×); (**b**) shape diagram of bending fracture (500×).

**Figure 15:**
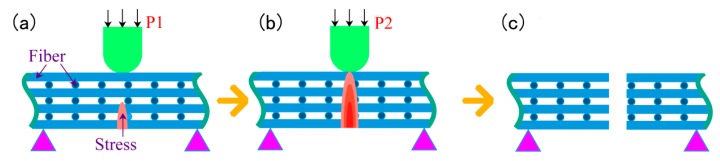
Schematic diagram of fracture process and stress distribution (0.9 MPa).

When the infiltration pressure is further increased to 0.9 MPa, the microstructure of the 2D-CFRP scanned by SEM is shown in Figure 14a and the bending fracture morphology is shown in Figure 14b. It can be seen from Figure 14a that fiber bundles have a certain amount of matrix distribution, and matrix content decreases significantly. At this time, the infiltration effect is not as good as prepared by 0.7 and 0.8 MPa. A closer look reveals a crack in the fiber bundle, which indicates that the infiltrated matrix is extruded by excessive infiltration pressure, so the local content of the matrix is less in the carbon fiber preform. It can be seen from Figure 14b that the overall content of the matrix decreases, the bending fracture is very neat, and there is no fiber is pulled out, which is a typical brittle fracture. It can also be seen from the figure that several cracks interlace and extend in the composite, and serious defects appear in the composite material at this time. When the 2D-CFRP is loaded, the fracture of the fiber is accompanied by the cracking of the matrix. The cracks in the matrix will quickly spread to the adjacent fibers, and the stress at the crack tip cannot be effectively dispersed, resulting in the overall brittle fracture of the material [20,21]. The schematic diagram of the fracture process and the stress distribution is shown in Figure 15.

By three-point bending test, when the infiltration pressure is 0.9 MPa, the bending strength of the 2D-CFRP is only 390 MPa. The bending property of 2D-CFRP decreased obviously. It is shown that excessive infiltration pressure not only reduces the infiltration effect, but also brings cracks to the 2D-CFRP, which has a negative effect on the infiltration effect and mechanical properties of the composite material.

### 3.2. Theoretical Calculation and Analysis and Summary of Experimental Results

In the above theoretical calculation, although the dynamic viscosity of the matrix takes into consideration, it is still based on an idealized infiltration model, and the air resistance and the frontend resistance are ignored. Moreover, the fiber arrangement in the form of 2D carbon fiber preform in practice is ununiform, so the calculation results are used as a theoretical reference and the actual infiltration pressure of the infiltration process should be greater than 0.478 MPa to ensure the infiltration. According to the experimental results of vacuum infiltration hot pressing molding, the infiltration pressure has an important influence on the infiltration effect and the morphologies of bending fracture. When the infiltration pressure is 0.5 MPa, the infiltration effect is insufficient, and the distribution of the matrix is unreasonable, so the bending strength is low, and the fibers are dispersed at the fracture. When the infiltration pressure is 0.7 MPa, the composite material has a good infiltration effect and a reasonable fracture appearance. Some fibers are pulled off and some are pulled out. Defects are effectively controlled, and the bending strength reaches 627 MPa. When the infiltration pressure reaches 0.9 MPa, the infiltration effect is significantly lower than the experimental optimal infiltration effect. At the same time, excessive infiltration pressure leads to cracks in the composite material, which results in the bending strength of the composite material is only 390 MPa and the bending fracture is relatively neat. 

## 4. Conclusions

(1) The critical infiltration pressure of matrix in a 2D carbon fiber preform is theoretically deduced and calculated. Under ideal conditions, the static and dynamic model values are 0.115 MPa and 0.478 MPa, respectively. Since there is no viscous resistance or gas pressure at the infiltration front during the calculation of the static model, the critical infiltration pressure obtained by statics is obviously less than the critical infiltration pressure obtained by dynamics. 

(2) 2D-CFRP with infiltration pressures of 0.5, 0.6, 0.7, 0.8, and 0.9 MPa were prepared by the vacuum infiltration hot pressing molding process. It was found that the infiltration pressure has an important influence on the infiltration effect, fracture morphology, and bending strength of 2D-CFRP.

(3) Low infiltration pressure will lead to insufficient infiltration of 2D-CFRP, so the fibers cannot effectively play the strengthening role and the bending strength of 2D-CFRP is low at this time. High infiltration pressure will lead to cracks in the 2D-CFRP and block the improvement of the bending strength of 2D-CFRP.When the infiltration pressure is 0.7 MPa, the composite material has an excellent infiltration effect and reasonable fracture morphology. And the bending strength reaches 627 MPa, which increases by 165% than the minimum bending strength.

(4) The fracture morphology of the prepared 2D-CFRP changes with the increase of infiltration pressure. When the infiltration pressure is low, the fracture is mainly scattered with fibers. When the infiltration pressure is appropriate, the fracture has both fiber pull out and fiber pull out, and the bending strength of prepared 2D-CFRP is the best. When the infiltration pressure is high, the fracture is flat.

## Figures and Tables

**Table 1:** Parameters by vacuum infiltration hot pressing molding process.

Curing Mixed Ratio/ Mass Ratio	Natural Curing Time /h	Pressure-Free Heating Curing Temperature/°C	Pressure-Free Heating Curing Time/min	Pressure Heating Curing Temperature/°C	Pressure Heating Curing Pressure/MPa	Pressure Heating Curing Time/min
5:1	4	80	20	50	0.5-0.9	3
